# In Vitro Effect of 8-Prenylnaringenin and Naringenin on Fibroblasts and Glioblastoma Cells-Cellular Accumulation and Cytotoxicity

**DOI:** 10.3390/molecules22071092

**Published:** 2017-06-30

**Authors:** Monika Stompor, Łukasz Uram, Rafał Podgórski

**Affiliations:** 1 Centre for Innovative Research in Medical and Natural Sciences, Faculty of Medicine, University of Rzeszów, Warzywna 1a, 35-310 Rzeszów, Poland; rpodgorski@ur.edu.pl; 2 Department of Biochemistry, Faculty of Medicine, University of Rzeszów, Warzywna 1a, 35-310 Rzeszów, Poland; 3 Faculty of Chemistry, Rzeszów University of Technology, 6 Powstańców Warszawy Ave, 35-959 Rzeszów, Poland; luram@prz.edu.pl

**Keywords:** 8-prenylnaringenin, naringenin, cellular accumulation, glioblastoma, cytotoxicity, confocal microscopy

## Abstract

Gliomas are one of the most aggressive and treatment-resistant types of human brain cancer. Identification and evaluation of anticancer properties of compounds found in plants, such as naringenin (N) and 8-prenylnaringenin (8PN), are among the most promising applications in glioma therapy. The prenyl group seems to be crucial to the anticancer activity of flavones, since it may lead to enhanced cell membrane targeting and thus increased intracellular activity. It should be noted that 8PN content in hop cones is 10 to 100 times lower compared to other flavonoids, such as xanthohumol. In the study presented, we used a simple method for the synthesis of 8PN from isoxanthohumol—*O*-demethylation, with a high yield of 97%. Cellular accumulation and cytotoxicity of naringenin and 8-prenylnaringenin in normal (BJ) and cancer cells (U-118 MG) was also examined. Obtained data indicated that 8-prenylnaringenin exhibited higher cytotoxicity against used cell lines than naringenin, and the effect of both flavones was stronger in U-118 MG cells than in normal fibroblasts. The anticancer properties of 8PN correlated with its significantly greater (37%) accumulation in glioblastoma cells than in normal fibroblasts. Additionally, naringenin demonstrated higher selectivity for glioblastoma cells, as it was over six times more toxic for cancer than normal cells. Our results provide evidence that examined prenylated and non-prenylated flavanones have different biological activities against normal and cancer cell lines, and this property may be useful in designing new anticancer drugs for glioblastoma therapy.

## 1. Introduction

A wide spectrum of biologically active flavonoids, including compounds from hops (*Humulus lupulus* L., *Cannabaceae*), have encouraged researchers to evaluate their possible applications in therapies for various diseases. This activity is based on molecular mechanisms involving mainly inhibition of cyclooxygenase (COX), and lipooxygenase (LOX) [1]. Common hops contain over 1000 chemical compounds, and is the main dietary source of prenylated flavonoids. The most important hop flavonoids include xanthohumol, isoxanthohumol, naringenin, 8-prenylnaringenin, and their derivatives.

Flavonoids have positive effects on the nervous system, improving blood flow to the brain due to formation of new blood vessels and growth of hippocampal neurons, which may improve memory processes. Such properties help to develop cognitive skills, important in Alzheimer disease therapy. In in vitro investigations with the use of hippocampal cells, naringenin promotes neurogenesis and stimulates growth of damaged neurons [2]. Although plants have been used for a long time in the therapy of various diseases, it is important to know the activity of individual compounds. According to research by Kuete et al., isolated naringenin was more efficient than the plant extract itself [3].

Due to their pro-health properties, hop flavonoids and their synthetic derivatives have been thoroughly studied. Xanthohumol, the most important chalcone, which makes up to 1% of the hop-cone dry weight, has many biological properties. Apart from strong antioxidant activity, it was proven to have antiviral [4], antimicrobial [5], and anti-inflammatory actions [6]. Moreover, in vitro study proved that xanthohumol inhibits formation of new blood vessels during carcinogenesis, and it has antiproliferative properties against various human cancer cell lines: breast (MCF-6, MCF-7, T47-D), colon (HT-29), ovarian (A-2780), and prostate [7,8,9,10,11,12].

There is some evidence that consumption of flavonoids considerably reduces the risk of some types of cancers—breast, colon, lung, prostate, and pancreatic [13]. Their mechanism of blocking DNA replication by inhibiting the activity of enzymes such as DNA polymerase II and topoisomerases I and II is known. Flavonoid compounds are also involved in cell cycle arrest, which results in blocking proliferation and inducing apoptosis of cancer cells. They are also capable of preventing oncogene activation by interactions with metabolic enzymes, for example, inhibition of cytochrome P450 enzymes, such as CYP1A1 and CYP1A2 [14].

In recent years there has also been great interest in 8-prenylnaringenin, a compound with a content in hop cones 10 to 100 times lower than that of xanthohumol [10]. 8-prenylnaringenin, a potential anticancer drug, demonstrates stronger affinity in vitro for the estrogen receptor ERα than coumestrol and genistein, compounds that have so far been considered as the most active flavonoids [15]. Movement of the prenyl substituent from C-8 to C-6 results in a loss of estrogenic activity. Additionally, other prenyl derivatives of naringenin present in hops, such as 6-prenylnaringenin, may play an important role in post-menopausal hormone-replacement therapy, being catalysts of regioselective estrogen hydroxylation, which reduces the risk of breast cancer [16].

Based on the diverse biological activity of flavonoids, their derivatives from hops may be potential therapeutic agents for the treatment of glioblastomas. Epidemiological studies have shown that the most common malignant glioblastoma multiforme is a resistant tumor of the central nervous system in humans, and effective strategies to inhibit its progression are particularly needed. The major problem in the treatment of glioblastomas is low penetration of anticancer drugs through the blood-brain barrier and their low toxicity against cancer cells [17]. This phenomenon is caused by the presence of multidrug resistance proteins, such as P-glycoprotein (P-gp), in blood-brain barrier (BBB) cells and in many glioblastoma cell lines [18,19].

A large number of studies have indicated that many flavonoids have an inhibitory activity on P-gp-mediated efflux [20]. Di Pietro et al. showed that prenylation of flavonoids increased their affinity for P-gp and their intracellular accumulation [21]. Although naringenin is able to cross the BBB in vitro and in in vivo models [22], and naringenin-induced apoptosis in glioma cells has been reported [23], the effect of 8-prenylnaringenin on glioma cells remains unclear. In the presented study, we examined cellular accumulation, distribution, and cytotoxicity of naringenin and 8-prenylnaringenin in U-118 MG cells, compared with normal human fibroblast cells.

## 2. Results and Discussion

### 2.1. Synthesis of 8-Prenylnaringenin

The most potent known phytoestrogen contained in hop cones (*Humulus lupulus*), and also in plants such as *Marshalia grandiflora* and *Sophora tomentosa*, is 8-prenylnaringenin. Because of its interesting biological properties, methods of obtaining 8-prenylnaringenin are being developed in addition to isolation protocols from natural sources. One of them is a four-step synthesis starting from naringenin, using a europium (III) complex and prenyl bromide in the Claisen–Cope rearrangement. However, this is a multi-step process, requiring protection of the 7-OH and 4’-OH hydroxyl groups, and therefore the reaction yield is moderate (48%) [24]. An alternative method described by Wilhelm and Wessjohann [25] involves demethylation of isoxanthohumol. The authors tested various demethylation agents and achieved a yield of 92%.

In our study, isoxanthohumol, obtained by alkaline isomerization of xanthohumol isolated from hops, was subjected to *O*-demethylation with ethereal solution of MgI_2_ to give 8-prenylnaringenin, with 97% yield. The obtained product was characterized spectroscopically. In the ^13^C-NMR analysis, the signals we ascribed to carbon atoms by using the NMR decoupling spectra and correlation 2D (two-dimensional) NMR spectra.

The analysis of the product spectral data (^1^H-NMR, ^13^C-NMR, IR, MS, and UV) led to the conclusion that the obtained isoxanthohumol derivative is without a methoxy group at C-5. In the HR ESI-MS (high-resolution electrospray ionisation mass spectrometry) spectrum, we observed the [M − H]^−^ ion of *m*/*z* = 339.1235, in accordance with the mass of [C_20_H_20_O_5_ − H]^−^ (339.1232). In the IR spectrum, there was no absorption band at 1275 cm^−1^, observed for the substrate (–OCH_3_ group).

In the low-field region of the ^1^H-NMR spectrum, there was a one-proton singlet at δ = 12.14 ppm of the free 5-OH group. The demethylation in the aliphatic region of the ^1^H-NMR of the product was evidenced by the absence of a three-proton singlet at δ = 3.73 ppm, present in the spectrum of isoxanthohumol. Due to the presence of the C-2 atom in 8-prenylnaringeninin, one can also see in the ^1^H-NMR spectrum signals of geminally coupled hydrogen atoms at C-3 (*J* = 17.0 Hz). In the DEPT 135° spectrum of the product, there are no signals of C=O (δ = 198.6 ppm), C-8 (δ = 109.3 ppm), C-9 (δ = 165.9 ppm), C-10 (δ = 104.3 ppm), and C-1’ (δ = 132.1 ppm), which indicates that they are the quaternary carbon atoms, whereas the signals of methylene carbon atoms C-1” and C-3 (δ = 23.2 ppm and δ = 44.4 ppm) were negative. The absorption band in the UV spectrum was observed at λ_max_ = 293.6 nm.

### 2.2. Cytotoxicity of Naringenin and 8-Prenylnaringenin

Prenylated hop compounds may play an important role in therapy of brain tumors. The major hop chalcone xanthohumol reduced cell viability and induced apoptosis in the U87 glioma cells and T98G cells of human glioblastoma multiforme [17].

**Figure 1:**
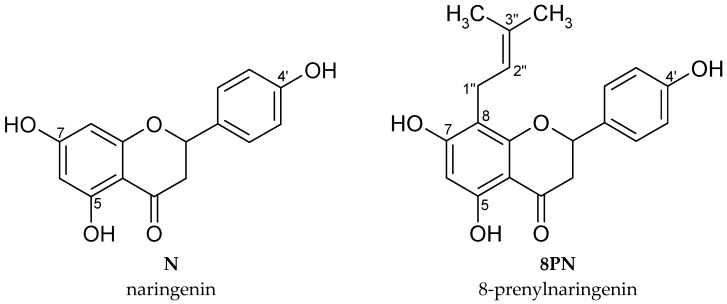
The chemical structures of the tested compounds.

Due to reports that substitution of the prenyl group increases the hydrophobicity of flavonoids and may led to its higher cellular accumulation [26] and in consequence, higher toxicity against cancer cells [27], the cytotoxicity of naringenin and 8-prenylnaringenin was compared in normal fibroblasts and U-118 MG glioma cells (Figure 1).

**Figure 2:**
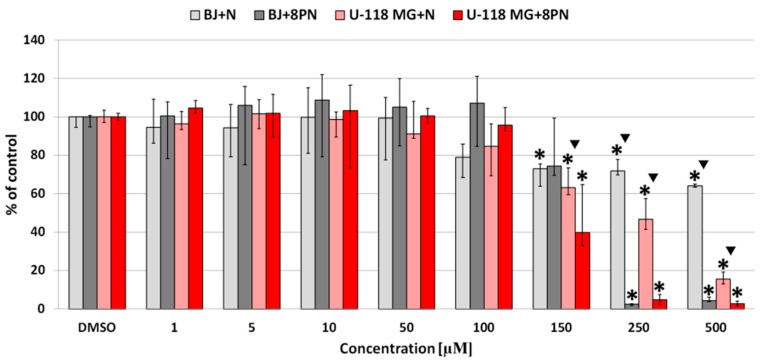
Effects of N and 8PN on the viability of normal human fibroblasts (BJ) and glioblastoma cells (U-118 MG) after 24 h treatment with flavonoids, assayed using the neutral red test. Results are presented as the median of triplicate assays from three independent experiments, and expressed as a percentage of the non-treated controls. The whiskers are lower (25%) and upper (75%) quartile ranges. * *p* > 0.05; Kruskal–Wallis test (against non-treated control), ▼ *p* < 0.05; Mann–Whitney U test (N against 8PN).

Neutral red cell viability assays showed that both flavonoids exerted stronger inhibitory effects on the cancer cell lines as compared with normal cells (Figure 2). At concentrations above 150 µM, 8PN was significantly more cytotoxic than naringenin for both cell lines, and higher toxicity was seen in U-118 MG glioblastoma cells compared with normal fibroblasts (Figure 2). Estimated IC_50_ for glioblastoma was found to be approximately 138 and 211 µM for 8-prenylnaringenin and naringenin, respectively, whereas in normal fibroblasts IC_50_ was 172 and 1090 µM for 8PN and N, respectively. It has to be pointed out that naringenin was more selective for glioblastoma cells as it was over 6 times more cytotoxic for cancer than normal cells, while higher inhibitory activity of 8-prenylnaringenin for both cell types was evident.

Similar results were obtained by other researchers. Tundis et al. [28] tested the antiproliferative effect of naringenin with nine cancer cells lines and one normal cell line after 48 h exposure. This flavonoid exhibited strong inhibitory effects against all cancer lines (IC_50_ from 2.2 to 178 µM) and very weak activity in normal human skin fibroblasts (142BR) (IC_50_ > 183 µM). Ayob et al. [29] estimated naringenin IC_50_ values for basal-B mammary carcinoma (MDA-MB-231), basal-A mammary carcinoma (MDA-MB-468), and Chinese hamster ovaries (CHO), with a similar range of values of 77 to 875.2 µM after 72 h treatment, using 3-(4,5-dimethylthiazol-2-yl)-2,5-diphenyl tetrazolium bromide (MTT) assay. This is comparable with our results.

The anticancer properties of 8-prenylnaringenin after 24 h treatment were measured with a colon cancer cell line (Caco-2) by Allsopp et al. [30]. They demonstrated that this prenylflavonoid showed no significant cytotoxicity against Caco-2 cells at concentrations up to 20 µM, with a 25% reduction of cell viability at 40 µM concentration. Toxicity of 8-prenylnaringenin was also evaluated by Tokalov et al. [31] with a resazurin-based assay. 8PN had no significant inhibitory effect on a human promyeloid leukemia cell line (HL-60) and a breast cancer cell line (MCF-7) at up to 50 µM concentration after 24 h incubation, but MCF-7 cells were clearly more resistant. This is in accordance with the results of our experiment. Our data confirm that the cytotoxicity of the investigated flavonoid compounds N and 8PN depended on cell type (normal/cancer), with 8PN always being more inhibitory than N. The in vitro activities of anticancer drugs against glioblastoma cell cultures was assessed by Jiang et al. [32]. Our results indicated that the tested compounds (N and 8PN) showed remarkably lower cytotoxic activity in comparison to positive compounds and drugs (IC_50_ between 0.019 and 20.14 µM).

### 2.3. Cellular Accumulation of Naringenin and 8-Prenylnaringenin

The cellular accumulation of N and 8PN was examined with the use of confocal microscopy on the basis of observations made by Mukai et al. [33] and Sudo et al. [34]. They used a fluorescence microscope to visualize cellular uptake of various flavonoids, due to their autofluorescence.

**Figure 3:**
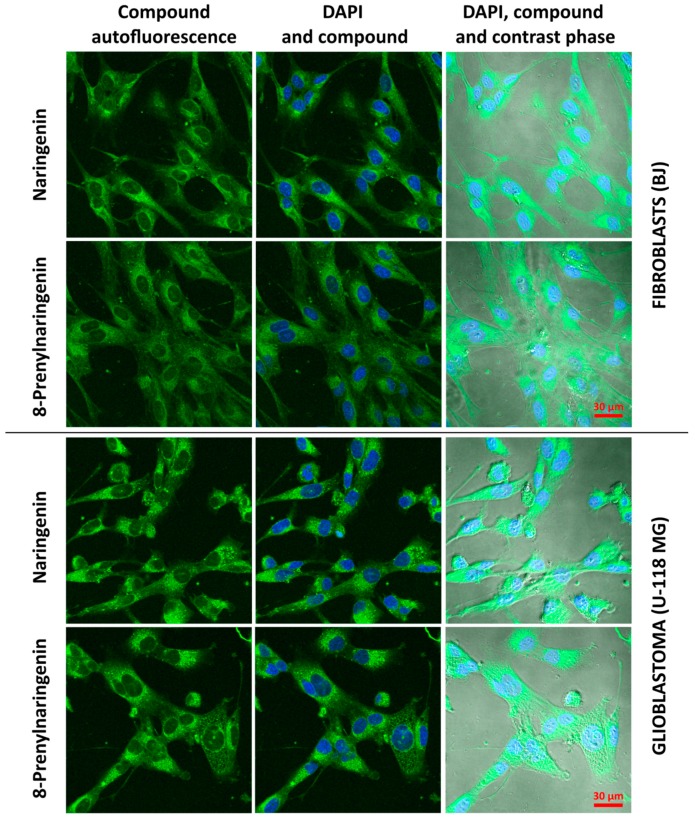
Accumulation and distribution of 10 µm naringenin and 8-prenylnaringenin in BJ and U-118 MG cells after 24 h incubation. Green signal was derived from flavonoid autofluorescence and blue signal from DAPI-stained nuclei. Scale bar 30 µm.

Confocal images of BJ and U-118 MG cells were obtained using 80% maximum energy laser excitation and high 80% gain because of the weak autofluorescence of both compounds. For this reason, cells were incubated with high, but non-toxic, 10 µM concentrations of both investigated flavonoids N and 8PN. Figure 3 demonstrates subcellular localization of both compounds after 24 h incubation with moderate penetration into nuclei.

N and 8PN were distributed evenly inside of the subcellular area of fibroblasts compared with glioblastoma cells, inside of which numerous vesicles with flavonoids were visible suggesting that the majority of internalized flavonoids may be accumulated inside subcellular organelles, such as lysosomes, golgi apparatus, endoplasmic reticuli, or mitochondria. It was reported that another flavonoid, quercetin, was binding to intracellular components and accumulated inside mitochondria [35]. Differences in the subcellular distribution of flavonoids and efficiency of accumulation/exclusion processes may lead to alterations in cell-line responses.

**Figure 4:**
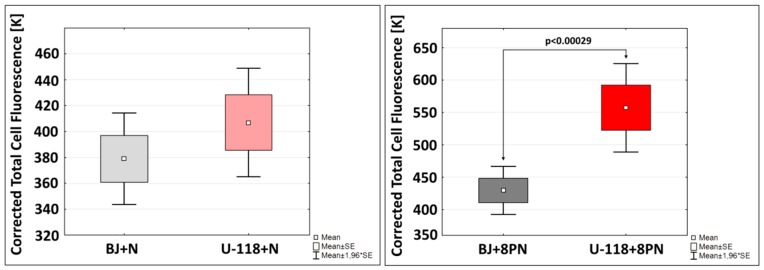
Accumulation of 10 µM naringenin or 8-prenylnaringenin in normal fibroblasts (BJ) and glioblastoma cells (U-118 MG) after 24 h treatment. Data presented as the mean of Corrected Total Cell Fluorescence. Error bars represent mean ± 1.96 * SE. Statistically significant differences are marked as arrows for *p* < 0.05 (Student’s *t*-test).

Accumulation and distribution of each of compound in investigated cell lines is presented on Figure 4.

Fluorescent signal intensity of naringenin in normal fibroblasts and glioblastoma cells was similar (Figure 4), whereas the accumulation of 8-prenylnaringenin in cancer cells was 37% higher than in normal cells. This phenomenon may be due to naringenin prenylation, which may generate higher uptake and accumulation in various cell types [36]. Obtained results are consistent with cytotoxicity patterns showing 8-prenylnaringenin as more toxic for glioblastoma cells than for fibroblasts. This phenomenon may be of greater importance at higher, toxic concentrations of both compounds. Moreover, the time of cell incubation with the examined compounds amounted to 24 h. In vivo studies have shown that prenylflavonoids remain in the blood for much longer periods than non-prenylated, so higher accumulation in the target tissue may be achieved by efficient cellular uptake [36]. Mukai et al. suggested that the biological potential of prenylflavonoids could be due to their greater absorption by the body and efficient accumulation in target tissues [37]. Therefore, cellular accumulation and selective toxicity against cancer cells in vivo may be stronger than in vitro, and this phenomenon requires further study in vivo. However, our in vitro data revealed that the presence of the isoprenyl group increases the biological activity of 8-prenylnaringenin, compared to non-substituted naringenin. These results indicate that addition of the prenyl group increases cellular uptake of 8PN and its anticancer activity in U-118 MG cells.

## 3. Materials and Methods

### 3.1. Reagents

Naringenin and other chemicals were purchased from Sigma-Aldrich (St. Louis, MO, USA). Organic solvents were dried and purified according to the standard procedures.

#### 8-Prenylnaringenin

Isolation of xanthohumol from spent hops and chemical cyclization of xanthohumol into isoxanthohumol was performed as described earlier [5,25]. 8-Prenylnaringenin was obtained by demethylation of isoxanthohumol in a two-step process, carried out under nitrogen atmosphere. At first, ethereal MgI_2_ solution was prepared in a 100 mL round-bottom flask. 0.33 g of magnesium was weighed and the flask was placed on a magnetic stirrer. Then, crystalline iodine (1.73 g, 3.39 mmoL) dissolved in anhydrous diethyl ether (60 mL) was added portionwise. The flask was tightly closed and the mixture was stirred in the dark for 4 h, until the colour disappeared.

In the second step 800.3 mg (2.26 mmoL) of isoxanthohumol was weighed and placed in a 250 mL 2-neck round bottom flask, which was immersed in a glycerin bath and placed on a magnetic stirrer, and 160 mL of tetrahydrofuran (THF) was added to it. Then, the clear solution of magnesium iodide etherate was added, through a long needle. The reaction was conducted at 65–72 °C, in the dark, for 20 h. The progress of the reaction was monitored by TLC, using chloroform–methanol 98:10 (*v*/*v*) as an eluent. When the substrate was fully consumed, the solvent was evaporated to a volume of 10 mL and then sat. NH_4_Cl (200 mL) was added. The resulting mixture was transferred to a separatory funnel and extracted with CH_2_Cl_2_ (3 × 100 mL). The combined organic layers were dried over Na_2_SO_4_, then filtered and evaporated off. The crude product was purified by flask chromatography using silica gel (eluent: CHCl_3_: MeOH 99:4 (*v*/*v*)) to give the pure product as yellow crystals.

8-Prenylnaringenin (yield 97%) m.p. = 192–193 °C lit. 193 °C [24] IR (KBr) cm^−1^: 3262, 1639, 1603, 1518, 1439, 1343, 1170, 1071, 832. ^1^H-NMR (500 MHz, acetone-*d_6_*) δ: 12.14 (1H, s, 5-OH), 9.52 (1H, s, 7-OH or 4’-OH), 8.50 (1H, s, 7-OH or 4’-OH), 7.41 (2H, d, *J* = 8.3 Hz, H-2’, H-6’), 6.90 (2H, d, *J* = 8.3 Hz, H-3’, H-5’), 6.02 (1H, s, H-6), 5.45 (1H, dd, *J* = 12.7 Hz, *J* = 2.8 Hz, H-2), 5.18 (1H, t, *J* = 7.0 Hz, H-2’’), 3.22 (2H, d, *J* = 7.0 Hz, H-1”), 3.14 (1H, dd, *J* = 17.0 Hz, *J* = 12.7 Hz, H-3 ax), 2.76 (1H, dd, *J* = 17.0 Hz, *J* = 2.8 Hz, H-3 eq), 1.60 (3H, s, H-4”), 1.59 (3H, s, H-5”). ^13^C-NMR (150 MHz, acetone-*d_6_*) δ: 198.6 (C=O), 165.9 (C-9), 163.9 (C-5), 162.1 (C-7), 159.6 (C-4’), 132.2 (C-3”), 132.1 (C-1’), 129.8 (C-2’, C-6’), 124.7 (C-2”), 117.1 (C-3’, C-5’), 109.3 (C-8), 104.3 (C-10), 97.4 (C-6), 80.7 (C-2), 44.4 (C-3), 26.9 (C-5”), 23.2 (C-1”), 18.9 (C-4”). HR ESI-MS *m*/*z*: 339.1235 [M − H]^−^; cald: 339.1232 [C_20_H_20_O_5_ − H]^−^. Anal. calcd. for C_20_H_20_O_5_: C 70.57; H 5.87. Found C 70.61; H 5.98. UV (MeOH) λ_max_: 338.7; 293.6 nm.

### 3.2. Cell Culture

Human glioblastoma U-118 MG cells (ATCC HTB-15) and normal human fibroblast BJ cells from a newborn male’s foreskin (ATCC-CRL-2522, population doubling level between 15 and 25, passage number between 5 and 9), Dulbecco’s Modified Eagle’s Medium (DMEM), Eagle’s minimum essential medium (EMEM), fetal bovine serum (FBS), and penicillin and streptomycin solution were obtained from the American Type Culture Collection (ATCC, Manassas, VA, USA). Tripsin-EDTA solution, phosphate-buffered saline (PBS) with and without magnesium and calcium ions, hydrocortisone, 0.33% neutral red solution (3-amino-m-dimethylamino-2-methyl-phenazine hydrochloride), and 0.4% trypan blue solution were provided by Sigma-Aldrich (St. Louis, MO, USA). DAPI (4′,6-diamidino-2-phenylindole, dihydrochloride) was purchased from Life Technologies (Carlsbad, CA, USA). Chamber culture slides were obtained from Nunc (Rochester, NY, USA), and all other cell culture sterile materials were purchased from Corning Incorporated (Corning, NY, USA).

Glioblastoma cells were cultured in DMEM, and normal human skin fibroblasts in EMEM media, containing 10% heat-inactivated FBS, 100 U/mL penicillin, and 100 µg/mL streptomycin at 37 °C, in 5% CO_2_, and at 95% humidity. Growth media were changed every 3 days and cells were passaged at 80% confluence after treatment with 0.25% trypsin-EDTA in PBS (calcium and magnesium free). Cell morphology was checked under a Nikon TE2000S Inverted Microscope (Tokyo, Japan) with phase contrast. Number and viability of cells were estimated by the trypan blue exclusion test using an Automatic Cell Counter TC20™ (Bio-Rad Laboratories, Hercules, CA, USA). All assays were performed in triplicate in three independent experiments.

### 3.3. Analytical Methods

The NMR spectra (^1^H-NMR, ^13^C-NMR, DEPT 135°, COSY, HMQC, HMBC) of 8-prenylnarinenin were recorded at 500 MHz on a Bruker Ultra Shield TM Plus instrument in acetone-*d*_6_. UV spectra (Cintra 303 spectrofotometer; GBC, Braeside, Australia) were recorded in methanol. HR ESI-MS was taken on a Bruker micrOTOF-Q spectrometer. Elemental analysis was done with an EA 1108, Carlo Erba analyzer. IR spectra were measured on a Mattson IR 300 Thermo-Nicolet spectrophotometer (Mattson, Warszawa, Poland).

Analytical TLC was carried out on silica gel G 60 F_254_ plates (Merck, Darmstadt, Germany) with a mixture of CH_3_Cl:MeOH in various ratios as developing systems. Compounds were detected by spraying the plates with 1% Ce(SO_4_)_2_ and 2% H_3_[P(Mo_3_O_10_)_4_] in 10% H_2_SO_4_, followed by heating to 120–200 °C. The product was separated by column chromatography using silica gel (SiO_2_, Kieselgel 60, 230–400 mesh, 40–63 µm, Merck).

### 3.4. Cytotoxicity Neutral Red (NR) Assay

The cytotoxic effect of N and 8PN was estimated by neutral red assay (NR), which is based on the ability of normal cells to incorporate and bind neutral red within lysosomes. NR cell assay is more sensitive than other commonly applied tests (tetrazolium salts, enzyme leakage, or protein contents) [38]. Both cell lines were seeded in flat-bottom 96-well plates at a density of 1 × 10^4^ cells/well and allowed to attach for 24 h before treatment. Working solutions of naringenin or 8-prenylnaringenin (1–500 µM) were prepared in culture media. The DMSO concentration was adjusted to 0.1% in all samples, which had no significant effect on treated cell lines (not shown). After 24 h exposure to naringenin or 8-prenylnaringenin, media were replaced by 100 μL of NR solution (2% of the culture medium volume) and cells were incubated for 1 h (U-118 MG) or 2 h (BJ), followed by washing (with PBS). 100 μL/well of fixative (50% ethanol, 49% H_2_O, and 1% glacial acetic acid) was added and plates were shaken until complete dye dissolution (300 rpm. 15 min, RT). Absorbance was measured at 540/620 nm with a microtiter plate reader (μQuant–BioTek, Winooski, VT, USA) against a blank sample (fixative without cells).

### 3.5. Confocal Microscopy

BJ and U-118 MG cells were seeded in 8-well chamber culture slides at a density of 4 × 10^4^/well. After 24 h incubation, culture media were replaced with a 10 µM solution of naringenin or 8-prenylnaringenin and cells were incubated for 24 h, followed by rinsing with PBS (3 times). Cells were fixed with 3.7% formaldehyde solution, washed with PBS (3 times), and cell nuclei were stained with 300 nM DAPI in PBS for 15 min. The images were collected with an Olympus FV10i confocal microscope (Olympus, Tokyo, Japan) under 60× magnification, using a 60× water immersion lens in blue (DAPI) and green (FITC) channel with the pinhole set to 1.0 confocal aperture in each channel (thickness of an optical section = 0.905 µm). Excitation and emission for flavonoids were set to 490 and 520 nm, respectively, according to results from Mukai et al. [33]. Confocal images were processed using ImageJ software with a bio-format plugin.

### 3.6. Cellular Accumulation of Naringenin and 8-Prenylnaringenin

Confocal microscopy images were analyzed using ImageJ software (US National Institute of Health, Bethesda, MD, USA). The total corrected cell fluorescence (TCCF) was measured using the procedure described by McCloy et al. [39]. Each cell was outlined and area, mean gray value, and integrated density parameters were measured, including background. Then, total corrected cell fluorescence was estimated according to the equation: TCCF = integrated density − (area of selected cell × mean fluorescence of background readings). The fluorescence of at least 90 cells was measured in each group.

### 3.7. Statistical Analysis

To estimate the differences between flavonoid-treated and non-treated control samples, a statistical analysis was performed using the nonparametric Kruskal–Wallis test and paired Mann–Whitney U-test, to evaluate the differences between cells treated with naringenin and 8-prenylnaringenin. To assess differences between degree of N and 8PN accumulation in cells, Student’s t-test was executed. *p* < 0.05 was considered as statistically significant. Calculations were performed using Statistica 12 software (StatSoft, Kraków, Poland).

## 4. Conclusions

Our study revealed that isoxanthohumol derivative 8-prenylnaringenin was more cytotoxic against both used cell lines than its structural analogue naringenin, and that both flavones inhibited glioblastoma cells stronger than normal fibroblasts. The evident anticancer properties of 8PN correlated with its significantly greater accumulation in U-118 MG cells than in BJ cells. Naringenin had higher selectivity for glioblastoma cells, being over 6 times more toxic for cancer than normal cells.

Evidence was provided that prenylated and non-prenylated isoflavones have different biological activities against normal and cancer cells, and this phenomenon may be useful in chemical synthesis to construct new, effective chemotherapeutic agents for glioblastoma.

Our results indicate that the activity of 8PN is relatively moderate, and that it is not likely to become a therapy in itself but could serve as a starting point for lead optimization.

To fully understand and assess the health effects of tested compounds, it is desirable to characterize their activity at the molecular level, and to compare their tissue distribution and subcellular localization in different cancer cell lines.
